# HyQue: evaluating hypotheses using Semantic Web technologies

**DOI:** 10.1186/2041-1480-2-S2-S3

**Published:** 2011-05-17

**Authors:** Alison Callahan, Michel Dumontier, Nigam H Shah

**Affiliations:** 1 Department of Biology, Carleton University, Ottawa, Ontario, Canada; 2 Stanford Center for Biomedical Informatics Research, Stanford University, Stanford California, USA

## Abstract

**Background:**

Key to the success of e-Science is the ability to computationally evaluate expert-composed hypotheses for validity against experimental data. Researchers face the challenge of collecting, evaluating and integrating large amounts of diverse information to compose and evaluate a hypothesis. Confronted with rapidly accumulating data, researchers currently do not have the software tools to undertake the required information integration tasks.

**Results:**

We present HyQue, a Semantic Web tool for querying scientific knowledge bases with the purpose of evaluating user submitted hypotheses. HyQue features a knowledge model to accommodate diverse hypotheses structured as events and represented using Semantic Web languages (RDF/OWL). Hypothesis validity is evaluated against experimental and literature-sourced evidence through a combination of SPARQL queries and evaluation rules. Inference over OWL ontologies (for type specifications, subclass assertions and parthood relations) and retrieval of facts stored as Bio2RDF linked data provide support for a given hypothesis. We evaluate hypotheses of varying levels of detail about the genetic network controlling galactose metabolism in *Saccharomyces cerevisiae* to demonstrate the feasibility of deploying such semantic computing tools over a growing body of structured knowledge in Bio2RDF.

**Conclusions:**

HyQue is a query-based hypothesis evaluation system that can currently evaluate hypotheses about the galactose metabolism in *S. cerevisiae*. Hypotheses as well as the supporting or refuting data are represented in RDF and directly linked to one another allowing scientists to browse from data to hypothesis and *vice versa.* HyQue hypotheses and data are available at http://semanticscience.org/projects/hyque.

## Background

With the advent of high-throughput technologies, there is an abundance of independent data such as gene and protein sequences, gene expression data, protein structures, protein interactions and annotations. At the same time, there is a shortage of tools and methods that can handle the task of integrating this information and allow a scientist to draw meaningful inferences. A significant amount of time and energy is spent in merely locating and retrieving information, rather than thinking about what that information means. There is an acute need to create *tools for thought*, which enable scientists to ask "what if" questions about a system, form explanations, and make and evaluate predictions [1]. It is clear that biomedical computing must evolve to address the growing disparity between the massive production of data and the small amounts of knowledge being extracted from this data.

Advancing knowledge in the biological sciences involves experimentally testing hypotheses and interpreting the results based on prior scientific work; as a result, research biologists must carry out the intensive tasks of collecting, evaluating and integrating large amounts of different kinds of information about organisms, cells, genes and proteins to generate a hypothesis about relationships that exist in the biological system under study. Once a hypothesis is generated, the next challenge is to evaluate the hypothesis with respect to what is already known and to design related experiments to test the hypothesis. Researchers face the challenge of seeking out new, relevant information online along with managing and interpreting volumes of experimental data.

The current methods in biomedical informatics that attempt to tackle the information integration task can be grouped into two coarse categories: 1) data-centric methods, where quantitative methods are used to spot trends and patterns in large datasets; and 2) knowledge-centric methods, where formal knowledge management methods are used to reason about a biological system to guide further exploration. Semantic Web technologies are emerging as the key enabling technology to bridge these two categories and begin to address the data-knowledge gap [2-4].

The Semantic Web facilitates knowledge representation, information sharing and data integration in a distributed, decentralized manner, through a standard set of languages and protocols. The Resource Description Framework (RDF) offers a simple but powerful representation mechanism for the Semantic Web, where facts are represented as collections of subject-predicate-object triples which can be typed by taxonomically organized vocabularies. Just as databases can be queried using SQL, RDF can be queried through the SPARQL Protocol and RDF Query Language (SPARQL) in a manner that allows access to distributed resources on the Web at query time. As a major linked data initiative, the Bio2RDF project exemplifies this approach by providing nearly 30 billion triples of life science data through a globally redundant and distributed set of SPARQL endpoints [5][6]. RDF specifies a highly flexible, but limited vocabulary in comparison to the Web Ontology Language (OWL2) which provides expressive elements such as existential and universal quantifiers, qualified cardinality restrictions, class constructors (union, disjunction), and object properties. Expressive OWL ontologies have been used to form the basis for developing reasoning-capable knowledge bases [7], including genomic knowledge found in the *Saccharomyces* Genome Database (SGD) [8] and the pharmacogenomics of depression as found in curated articles highlighted by the Pharmacogenomics Knowledge Base [9].

Our previous research towards bridging the data-knowledge gap led to the development of the HyBrow hypothesis browser [10], which is a prototype system for formalizing and testing working hypotheses about gene regulation pathways. To demonstrate the proof-of-concept of these methods, we used the yeast galactose metabolic and regulatory network (GAL) [11]. HyBrow allows a user to express hypotheses about galactose metabolic regulation in yeast and to test their hypotheses against a small knowledge base about the GAL system, including knowledge derived from the literature as well as promoter binding site and gene expression data [10].

Related research efforts include work in generating and formally representing biological hypotheses and in formally evaluating hypotheses using experimental data. Tran *et al.*[12,13] developed a formal representation for hypotheses about cellular signalling, and demonstrated the use of abductive reasoning to formulate hypotheses about p53 regulation of tumour suppression. An important contribution of this work is the use of a representation language that facilitates non-monotonic reasoning, which was not possible with previous research in biological hypothesis representation such as HYPGENE [14][15], HinCyc [16] and GenePath [17]. Adam the Robot Scientist [18], a combination system for carrying out automated wet lab experiments and reasoning over hypothesis spaces, uses abductive reasoning to develop hypotheses and deductive reasoning to test them. The Robot Scientist also uses a formal representation of hypotheses [19]. Tari *et al.*[20] developed a system that combines natural language processing of Medline abstracts with a formal representation for drug-drug interactions (DDIs) in order to identify potentially undiscovered DDIs. Their system allows for the formulation of hypothetical drug interactions and subsequent evaluation using drug interaction statements extracted from Medline abstracts and DrugBank. Riboweb [21][22] is a similar system that allowed the representation of scientific data about ribosomes in a formal machine understandable manner, and allowed users to evaluate models of ribosomes. A more general system for evaluating hypotheses was developed by Gershman et al. [23], using Bayesian reasoning to evaluate hypotheses in the context of known, but incomplete data. Motivated by the HyBrow prototype system and the application of knowledge representation and Semantic Web technologies to life science problems, we present HyQue—a Semantic Web tool for querying scientific knowledge bases for the purpose of evaluating user submitted hypotheses. HyQue features a flexible knowledge model to accommodate diverse hypotheses structured as events and represented using Semantic Web languages (RDF/OWL). Hypothesis validity is evaluated according to experimental and literature-sourced evidence through a unique combination of automatically generated SPARQL queries and domain specific evaluation rules. Inference over OWL ontologies (for type specifications, subclass assertions and parthood relations) and retrieval of facts stored as Bio2RDF linked data provide support for a given hypothesis. Unlike HyBrow, this system is capable of executing queries where participating entities or event types are underspecified or completely unspecified, thus opening the door to a significantly wider range of hypothesis evaluation. In addition, hypotheses as well as the supporting or refuting data are represented in RDF and thus directly linked to one another allowing browsing from data to hypothesis and *vice versa.*

The work presented here is intended to demonstrate a framework for automatically performing information integration for the purpose of hypothesis evaluation. HyQue is applied to the GAL gene network domain as an exemplar, to describe the kinds of hypotheses and questions that can be posed over knowledge about such as system. This example application is relevant because it demonstrates a formal and computational evaluation of the kinds of hypotheses and queries that are typically of interest to molecular biologists and which often require significant manual effort to answer. The application of HyQue to the GAL network in *Saccharomyces cerevisiae* is further relevant because the galactose network is a pathway that has been the focus of intensive research to fully elucidate its genetic and molecular regulation. Focusing our effort on representing the results of this well-studied research area in a consistent and machine understandable manner and performing question answering as well as hypotheses evaluation allows us to demonstrate the capabilities of the HyQue infrastructure in a data-rich environment.

## Methods

PHP scripts were developed for converting *S. cerevisiae* GAL gene network knowledge to a linked data format and also for performing hypothesis evaluation, using ARC2 for RDF processing and SPARQL result processing. Information about the HyQue project, including data and SPARQL endpoint(s) is available at the HyQue site, http://semanticscience.org/projects/hyque.

### Knowledge Base Design and Creation

A key component of the HyQue system is the **HyQue Knowledge Base (HKB)** over which hypothesis-evaluating queries can be posed. The HKB was constructed from two main sources:

1. The manually curated *S. cerevisiae* galactose gene network data from [10] which includes data about the following seven event types:

1. protein-protein binding

2. protein-nucleic acid binding

3. molecular activation

4. molecular inhibition

5. gene induction

6. gene repression

7. transport

These event types describe the majority of cellular events and interactions in the Gene Ontology (except catalysis). This data was converted to RDF (N3 serialization) using Bio2RDF identifiers and typed with the following public biomedical ontologies:

**- Gene Ontology (GO)**: cellular components, events (*e.g.* ‘nucleus’, ‘positive regulation of gene expression’)

**- Evidence Codes Ontology (ECO)**: the type of evidence supporting an event (*e.g.* 'electronic annotation', 'direct assay')

**- Sequence Ontology (SO)**: event participants (*e.g.* 'gene')

**- Chemical Entities of Biological Interest (CHEBI)Ontology**: event participants (*e.g.* 'protein', 'galactose')

2. *S. cerevisiae* gene and gene product information from the *Saccharomyces* Genome Database (SGD). We extended the yOWL knowledge base [8] to provide more granular information about *S. cerevisiae* genes and gene products. In particular, yOWL now assigns Sequence Ontology (SO) terms to chromosomal features and distinguishes gene products from genes, unlike the SGD. Gene products now have identifiers created by appending ”gp” to the SGD identifier (e.g. sgd:S000002430 → sgd:S000002430gp) and are typed as ‘protein’ (CHEBI:36080) or ‘RNA’ (CHEBI:33697) as appropriate. Gene products are then associated with function, localization, processes, complexes, and physical interactions. If the gene product corresponds to a protein, then we make the gene product identifier equivalent (using owl:sameAs) with the derivative preferred and standard names (e.g. YDR023W → Ydr023wp; SES1 → Ses1p), along with protein identifiers from other databases. Thus, genes are associated only with information about the gene products they encode, chromosomal location, genetic interactions, phenotypic experiments, and corresponding/identical genes described in other databases.

### Querying the HKB to evaluate hypotheses

#### Representing hypotheses in HyQue

Hypotheses are first formulated using the HyQue Hypothesis Ontology. A *hypothesis* consists of one or more *events* in which the participating entities (and, optionally, the physical location and genetic perturbation context) are specified. Complex hypotheses can be specified by logically combining the events using the AND, OR and XOR operators, potentially leading to nested events *e.g.* A *AND* (B *OR* C). The AND operator indicates that multiple events must be satisfied for the hypothesis to be satisfied. In contrast, the OR operator simply indicates that if any of the specified events are true, they will satisfy the hypothesis. This may be appropriate when multiple mechanisms are possible, such as a phenotype resulting from the activation of more than one pathway. Finally, XOR operator stipulates that only one of the events must be true, else they are both false. For instance, one might hypothesize that a protein is either involved in gene regulation either as a nuclear-bound transcription factor or as a membrane-bound signal receptor, and clearly both cannot be true.

#### Evaluating hypotheses using HyQue

Hypotheses are evaluated by identifying relevant experimental data from the HyQue Knowledge Base. First, a SPARQL *construct* query is automatically generated from the input hypothesis using a query template that corresponds to one of the 7 defined event types. Second, the SPARQL query is executed against the HKB (currently a Virtuoso triple store) and the results are captured as an RDF graph. Finally, the RDF graph is analyzed by executing the scoring rules to calculate a set of scores for each part of the hypothesis, followed by determining the overall score for the entire hypothesis.

Execution of the SPARQL queries results in a set of triples that are processed to identify experimental evidence that best supports the hypothesis. Each hypothesized event is independently evaluated in order to quantify the degree of support it lends to the hypothesis. HyQue combines the individual event scores based on the operators between events (AND – add scores; OR – select maximum score; XOR – use single event score). Since events may be nested *e.g.* A *AND* (B *OR* C), nested event operators are evaluated first, followed by outermost operators, from which the final score is obtained. In cases where there is no or insufficient information to either support or refute a hypothesized event or set of events, the system declares these as 'undecidable', thereby rendering a conjunctive clause with an undecidable event as undecidable.

Event scores are determined from scoring rules. A scoring rule assesses deviations from the 'ideal' event that would provide maximum experimental support to the hypothesized event; they are based on expert knowledge. Deviations reduce the score assigned to the hypothesized event. Explicit contradictions, such as data indicating that a hypothesized event does *not* occur, receive a higher penalty than other deviations, such as data indicating that an event occurs in a different cellular location than that specified in the hypothesis. The rules used by HyQue for evaluating experimental GAL system data are primarily based on those developed for HyBrow, but modified to use knowledge represented in relevant bio-ontologies (GO, CHEBI *etc*.). The score for each event is represented as a fraction of the maximum score possible in order to normalize the score across event types with differing amounts of information available in the HKB. In the case of multiple data supporting an event, the data which contributes the maximum score is selected and linked to the hypothesized event.

To illustrate the nature of rule sets, consider the ‘induce’ rule set, based on [10]. Matching annotations for molecule type, functionality and localization increase the score while non-matching annotations decrease the score:

1. If actor is of type ‘protein’ (CHEBI: 36080) or ‘RNA’ (CHEBI:33697) add 1 to score; else subtract 1

2. If target is of type ‘gene’ (SO:0000236) add 1 to score; else subtract 1

3. If actor has function ‘transcription factor activity’ (GO:0003702) add 1 to score

4. If event location is ‘nucleus’ (GO:0005634) add 1 to score; else subtract 1

5. If the relationship between the actors is ‘induce’ add 1 to score; else subtract 1

6. If the hypothesized event is negated in the HKB, subtract 2 from score

While we have not done so for the ‘induce’ rule set (because the data does not support this annotation), the source of experimental evidence may also be specified in the scoring rule using the evidence code ontology (ECO). For example, data collected from a wet-lab experiment may be considered to have more weight than a database annotation whose source cannot be verified, and this can be incorporated into the hypothesis-evaluation process. Evidence types with greater validity contribute more to a score than weaker evidence types.

#### Representing hypothesis evaluations

Hypothesis evaluations are also specified using the HyQue Hypothesis Ontology. Each instance of HyQue *evaluation data* is *about* a *hypothesis*, and *has part* an overall *hypothesis score*. The overall *hypothesis score* has as its parts *combined event scores* and/or *maximum event scores*. These scores are typed depending on how they are calculated, *e.g.* a *maximum event score* is a score calculated by selecting the highest value of a set of possible *event scoresabout events* that are related by the OR operator, while a *combined event score* is a score calculated by combining the *event scores* of several events related by the AND operator. *Combined* or *maximum event scores* have individual *event scores* as their parts. *Event scores* are *derived* from *rule scores*, which have as their parts other *rule scores* that correspond to individual rules. Finally, *rule scores* are *about* the data upon which the rule was executed. In this way, hypotheses are linked to both the rules used to evaluate them and the data upon which their evaluation score is based. The HyQue namespace is used for all type declarations relevant to evaluations such as score types and hypotheses. The HyQue Data namespace is used for all data resulting from and contributing to evaluation – actual score values, evaluation instances and experimental data.

## Results

### Hypothesis evaluation

HyQue is currently implemented over a prototype knowledge base (HKB) consisting of information about the galactose metabolism gene network. Using this prototype knowledge base, HyQue can evaluate seven common biochemical events (protein-protein interactions, protein-nucleic acid interactions, activation, inhibition, gene induction, gene repression and transport) under specific conditions and in specific cellular environments. We have formulated a series of hypotheses about these types of events. We present two of these hypotheses with their evaluations here, and have made the remainder available at the HyQue website, http://semanticscience.org/projects/hyque.

The first hypothesis considers the induction of gene expression by the proteins Gal3p, Gal4p and Gal80p, which are known to play a regulatory role with respect to the genes that control the conversion of galactose to glucose-6-phosphate [11]. The hypothesis is composed of three parts of increasing complexity, all connected by the ‘OR’ relation, which indicates that they can be evaluated independently. The hypothesis is expressed in natural language (with event numbers *e_n_* for reference) and then step-wise evaluated, with the evaluation rationale explained.

#### Hypothesis

*e_1_* (Gal4p induces expression of GAL1)

OR

*e_2_* (Gal3p induces expression of GAL2

*e_3_AND* Gal4p induces expression of GAL7)

OR

*e_4_* (Gal4p induces expression of GAL7

*e_5_AND* Gal80p inhibits production of Gal4p

when GAL3 is over-expressed

*e_6_* AND Gal80p induces expression of GAL7)

The first event, *e_1_*, describes the induction of GAL1 gene expression by Gal4p and is therefore an event of type ‘induce’. The event is evaluated as follows, using the ‘induce’ rule set (specified in the Methods) over the data obtained from a ‘induce’ specific SPARQL query to the HKB.

1. Actor of type ‘protein’: yes -> +1

2. Target of type ‘gene’: yes -> +1

3. Actor has function ‘transcription factor activity’: no -> 0

4. Event location is ‘nucleus’: yes -> +1

5. Logical operator is ‘induce’: yes -> +1

6. Event negated in published literature: no -> 0

Thus, the *e_1_* event obtains 4 out of a maximum of 5 points, and receives a score of 0.8. Events *e_2_*, *e_3_*, and *e_4_* are also ‘induce’ events and are evaluated using the ‘induce’ rule set, each obtaining a score of 0.8. However, *e_5_* is 'undecidable' because the HKB does not contain data that states that Gal80p inhibits Gal4p when GAL3 is over-expressed. Since *e_5_* is undecidable and *e_4_*, *e_5_* and *e_6_* are related by the ‘AND’ operator, this third entire event set is deemed undecidable. Thus, the overall hypothesis score is based on the scores for the event set consisting only of *e_1_*, and the event set consisting of *e_2_* + *e_3_*. Since the event set composed of *e_2_* + *e_3_* receives the highest score of 1.6 (0.8+0.8), the final hypothesis score is 1.6. This score also indicates that the hypothesized events *e_2_* + *e_3_* have the strongest experimental support.

Another common biological phenomenon that can be evaluated by HyQue is the intracellular transport of molecules. The accepted model of the GAL system indicates that protein products of the GAL2 gene are responsible for transporting galactose into cells. This can be represented as a HyQue hypothesis (Table 1) for evaluation. Evidence for this transport event is obtained from HKB using the ‘transport’ SPARQL query template and subsequently evaluated using the ‘transport’ rule set.

**Table 1:** RDF representation of a hypothesis about galactose transport


	

**Figure 1:**
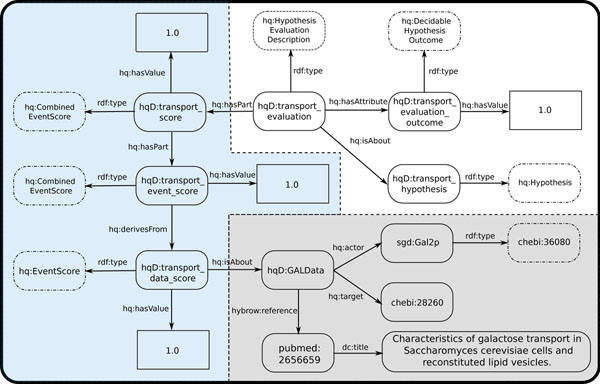
**A representation of the RDF output describing the evaluation of the galactose transport hypothesis**. The figure shows how evaluation results are linked to the experimental data used to support the hypothesis. Rounded rectangles with solid lines are class instances; rounded rectangles with dotted lines are ontology classes; rectangles are literals. The grey section shows the experimental GAL system data (from the HKB) used to evaluate the hypothesis, including source literature. The blue section shows the scores resulting from evaluating the galactose transport hypothesis. The white section shows the summarized evaluation results (decidable, with the overall score). ‘hq:’ is the HyQue namespace; ‘hqD:’ is the HyQue Data namespace; ‘sgd:’ is the *Saccharomyces* Gene Database namespace; ‘chebi:’ is the CHEBI namespace.

Partial results (represented as RDF by HyQue) of the HyQue evaluation are illustrated in Figure 1. The hypothesized transport of galactose by GAL2 protein product has strong experimental support [24], resulting in a hypothesis score of 1. Further exploration of the data used to evaluate the hypothesis may be accessed at its corresponding Bio2RDF URI: http://bio2rdf.org/hybrow:4730296b268ba03421d4a23ae449c8d9.

This example demonstrates that the methodology developed for hypothesis evaluations in HyQue correctly interprets experimental data and generates evaluations that are consistent with the current understanding of the GAL system as published in the literature. Four additional GAL system hypotheses representative of other commonly investigated cellular events and their evaluations are also described at the project web site.

### Hypothesis evaluation results

Figure 1 shows details of how the evaluation results for the galactose transport hypothesis are linked to experimental data from the HyQue Knowledge Base. The overall evaluation result for the hypothesis (in white) is linked to the scoring of the hypothesis (in blue) by the ‘*has part*’ relation. The score is further broken down into the partial scores, which are composed of individual event scores. Scores are related to experimental data (in grey) using the relation ‘*is about*’. Experimental data is composed of results from the literature which describe interactions and have actors and targets that are typed, in this example, using the CHEBI ontology. It can be seen that a user can browse from the hypothesis instance to the GAL data and *vice versa*.

### Question answering using HyQue

In addition to evaluating hypotheses as described above, it is also possible to pose queries to the HKB that represent questions about single events or entities that meet a set of specified criteria. For example, consider the query for retrieving all proteins that bind to the promoter region of the GAL1 gene. In this case the protein entities are unknown in advance and are instead specified in the corresponding SPARQL query as an unbound variable (see Table 2). Similarly, the context in which the promoter binding events occur is unknown, but can be retrieved by assigning it to an unbound variable. The result for such a query is shown in Table 3. It can be seen that three entities are known to bind to the promoter of the GAL1 gene in wild-type yeast. As can also be seen in Table 3, for certain event types such as “promoter binding”, the type of the evidence that supports a hypothesized event differs. For example, some data in the HKB is evidenced by the results of experimental assays while other data is evidenced by annotations in other databases. As described in the Methods, these two types of evidence have different 'strengths' in the context of determining the likelihood of a hypothesized event.

**Table 2:** SPARQL query for proteins that bind to the GAL1 promoter region




**Table 3:** Results of a SPARQL query for proteins that bind to the GAL1 promoter region

Actor	Target	Perturbation Context	Evidence Type
http://bio2rdf.org/sgd:Mig1p	http://bio2rdf.org/sgd:GAL1	wt	
http://bio2rdf.org/sgd:Spt15p	http://bio2rdf.org/sgd:GAL1	wt	eco:0000008
http://bio2rdf.org/sgd:Gal4p	http://bio2rdf.org/sgd:GAL1	wt	

### HyQue Hypothesis Generation Interface

While Semantic Web technologies such as RDF, OWL and SPARQL enable programmatic methods to create or query data, the manual composition of hypotheses in RDF is challenging. In order to facilitate the composition of hypotheses by biologists and biochemists, we have developed a prototype tool for generating hypotheses, available at http://semanticscience.org/projects/hyque/build.html. The interface makes it possible to define individual events and combine them to express a more sophisticated hypothesis. The resulting RDF can then be used as input to HyQue.

## Discussion

### From HyBrow to HyQue: Steps forward

A central aspect of HyQue is that the input (hypothesis), background knowledge (ontologies), data (conditional events, genes, proteins, *etc*.), queries, and outputs (scores) may be specified using Semantic Web technologies (OWL, RDF, SPARQL). Thus, it becomes possible to seamlessly navigate these heterogeneous different data sources when they are identified by unique dereferenceable URIs. Moreover, with hypotheses and their evaluations as Linked Data, it becomes possible to not only explore which data serve as the basis for hypothesis scores, but also which data serve as evidence for evaluated hypotheses. Users can explore the underlying evidence for their submitted hypotheses or identify which hypotheses are supported by some given data of interest, which was not possible in the HyBrow prototype.

SPARQL is a powerful graph-based language that enables the querying of specific individuals or a collection of individuals by reasoning about their type or by a set of relations or attributes that the members must hold. Hence, individual entities may be, but need not be explicitly specified in the query, and this feature alone differentiates the approach from that of the hard-coded rules in HyBrow [10]. Moreover, simple reasoning may be invoked to identify individuals that are sub-sets of the specified types. For example, one can ask for all proteins that bind to the promoter region of the GAL1 gene, with an optional clause for some associated conditions, such as the presence of galactose. In this case, the query is specified as a restriction on the type of entity participating in an event, rather than on the specific instance. Even more broadly, a query to identify ‘RNA’ will not only identify those molecules that are annotated as RNA, but will also identify those individuals that belong to more specific types such as ‘messenger RNA’, provided that sub-types are correctly specified in the type hierarchy of some ontology (*e.g.* the Sequence Ontology).

Lastly, and perhaps most importantly, the hypotheses presented here would require significant manual effort by a scientist to evaluate using existing experimental data, because of the complex coordination of data required. As the number of clauses in a hypothesis increases, the number of ways in which they can be combined and evaluated also quickly increases. A scientist undertaking the task of manual hypothesis evaluation would have to evaluate each combination in isolation and determine which represented the best support for the hypothesis. HyQue is able to automatically combine user-specified clauses with an event-specific template SPARQL query and then evaluate the query results in light of the type(s) of events contained in the hypothesis, where the event types are derived from shared bio-ontologies such as the Gene Ontology. The scientist may then explore how experimental data was used by HyQue to provide support/non-support for the submitted hypothesis by browsing the linked data (Figure 1), a high-level task more appropriately left to investigators.

### HyQue and related systems

In the HyQue system we specify the biologist’s notion of a hypothesis about a biological system using a formal language that represents the entities participating in a biological system (such as a pathway) and the relationships among them, in a manner similar to that adopted by HyBrow [25,26]. By using a formal language comprised of biological entities and explicit relationships among them, we can create an interpretation—a hypothesis that instantiates certain relationships between entities—that depicts a given biological system in a manner that satisfies the data at hand. This interpretation may then be evaluated by reasoning about its parts, as described in the Methods.

In contrast, the formalized hypothesis representation [19] used by Adam the Robot Scientist [18] is minimal in terms of semantics, and does not play a key role in the reasoning carried out by the system. It is used to express subclass relationships between different levels of hypotheses, but not to evaluate the hypotheses themselves. Instead, the Robot Scientist uses abductive reasoning to identify hypothesis spaces and to develop hypotheses as explanations for observed results from the experiments it carries out. Indeed, the primary difference between the Robot Scientist and HyQue is that the Robot Scientist abductively generates hypotheses based on its knowledge base while HyQue uses experimental results to evaluate *user-generated* hypotheses and identify statements of support or contradiction for them.

While other approaches [12,13] to hypothesis formulation and evaluation enable complex reasoning, they do not support easy access by scientists or publication on the Web as is possible with HyQue through its implementation of Semantic Web technologies. Indeed, the potential offered by the Semantic Web is particularly promising for applications such as HyQue, which emphasize re-use of data, explicit descriptions of assertions and knowledge, and online availability.

### Linked data, nanopublications and the future of HyQue

Using linked open data creates new opportunities for knowledge discovery in terms of ease of access, de-centralized publication of data, and new inferences from the integration of distributed data. The Semantic Web provides a set of standards for knowledge management and reduces the high barrier in normalizing heterogeneous data to a common and machine interpretable syntax and semantics. Perhaps more significant is that because linked data can be independently curated and published, it provides a scalable framework to vastly increase the knowledge space for evaluating hypotheses. Moreover, due to the continuous expansion of Bio2RDF (now serving over 40 billion triples) through user contributed content, the total amount of information available is expected to increase considerably in the coming years; which will increase the scope of hypothesis testing to new domains, such as pharmacogenomics or drug discovery.

**Figure 2:**
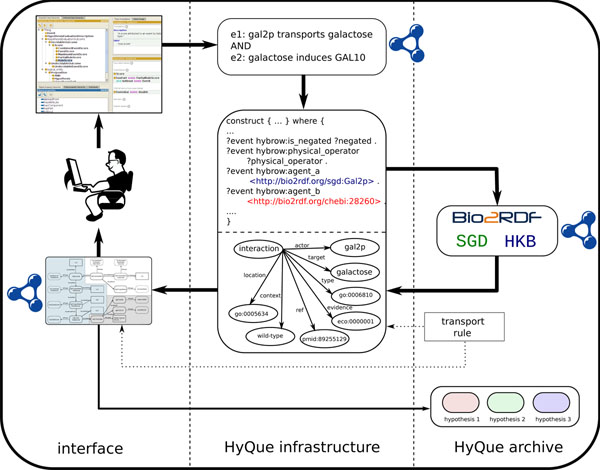
**A schematic of the future HyQue platform**. A user formulates a hypothesis using terms from the hypothesis ontology (top left), which is converted to a corresponding SPARQL query (upper center). Evaluation rules are applied the data retrieved by the SPARQL query (lower center) to generate scores of support and contradiction. The user is presented with an overview of the data used to evaluate the hypothesis along with support/contradict scores (bottom left). Hypotheses, data and evaluations are contributed to the HyQue archive.

Using event specific SPARQL *construct* statements for retrieving hypothesis evaluation results makes hypothesis evaluation more efficient than that originally described in [27] in that any information retrieved by a SPARQL query that is not required for evaluating the hypothesis is not passed to the evaluation engine. In addition, the use of *construct* statements to generate evaluation results as RDF means that the underlying data and scores of hypothesis evaluations can be directly linked to the user-generated hypotheses and the Bio2RDF linked data used as the basis for the evaluation. This approach for representing experimental and literature-derived knowledge as linked data provides functionality for exploring hypotheses and the data that supports them. Because linked data provides a reciprocal relationship so that one can explore incoming and outgoing links, scientists browsing the raw linked data may become aware of theories submitted by others that are supported by the experimental data of interest to them. HyQue's hypothesis-based queries can also be used to track the types of data scientists are using to support their hypotheses. This could lead to a system in which 'well-used' but not exhaustively validated data is flagged for further experimental evaluation to increase its credibility for providing hypothesis support. Conversely, hypotheses posed to HyQue that have no candidate supporting data can act as a seed for novel biological studies. We outline such a system in Figure 2.

As scientists use and contribute to HyQue, individual facts in the HyQue Knowledge Base also have the potential to become cardinal assertions, based on their usage in supporting or refuting hypotheses. Cardinal assertions and nanopublications [28] are related concepts that have been developed by the Concept Web Alliance to envision a future where individual statements can be published (nanopublications) and gain support based on the usage of those statements by the community, thus becoming cardinal assertions [29]. Such assertions would be expressed in RDF, potentially as named graphs. Key to the credibility of cardinal assertions and nanopublications is the related expression of their context, something that HyQue achieves in its event-based framework that describes interacting entities and also the *conditions* under which they interact (or do not interact).

### Scoring and its effect on hypothesis evaluation in HyQue

The rule sets currently used by HyQue are empirical, and based on previously existing rules in the HyBrow system. In translating these rules for HyQue and using them to score hypotheses, a number of discussion points about the HyQue scoring system have come to light. The current HyQue scoring system favours hypotheses that are composed of multiple events, because if those events are partially supported by existing experimental data in the HKB, the hypothesis will receive a higher score than if a hypothesis consisting of a single event was fully supported. On the other hand, hypotheses that are composed of multiple events such as the final part of the hypothesis described in the *Hypothesis evaluation* section, are more likely to be ‘undecidable’ or to have less support because they require more experimental data to be evaluated. An alternative scoring system based on determining the mean of event scores as opposed to their sum would have the opposite effect, where simpler hypotheses would be favoured. In a scenario where scientists pose competing hypotheses and where the score assigned to the hypothesis by HyQue plays a role in deciding whether the hypothesis is experimentally tested and/or accepted by the community, decisions about the scoring system will become increasingly important. Currently we are far from that state and intend to explore the effect of alternative scoring schemes on hypothesis evaluation in future work. It may also be the case that one rule set and scoring system used to evaluate hypotheses by one scientist may not be deemed appropriate by another scientist, thus requiring additional rule sets to take into account multiple research contexts.

### Representing negation in HyQue

In HyQue, events that are known not to occur are captured by asserting FALSE for the boolean “is negated” datatype property. While languages such as OWL2 have built-in expressions for negation that can be used in automated reasoning, the problem is that OWL is a monotonic language and the knowledge base cannot have both a statement that is asserted to be true and false at the same time. To reason over an OWL knowledge base containing contradicting statements would require that the contradictions be removed or the corresponding ontology be repaired. Thus, the approach taken here allows statements about the existence or lack of existence of an event having identical conditions to be represented and subsequently queried.

### Scalability

Using RDF and related Semantic Web technologies facilitates constant updating of the HyQue Knowledge Base. New data and facts can always be added to the existing system, without having to change how previously contributed information is represented or stored. This has been identified as a key property of systems for knowledge representation and question answering in this domain [2,13]. The current version of HyQue uses a knowledge base that includes the entirety of the SGD data from Bio2RDF, the Gene Ontology, ChEBI, ECO and SO, in addition to data specific to the GAL system. The HKB is hosted using the OpenLink Virtuoso triple store platform. As the underlying knowledge bases increase in size as well as complexity, as the evaluation approaches become more customizable, and as the system becomes applicable to multiple domains (see Future Work), HyQue’s performance will largely depend on the performance of triple store software employed. In addition to simply addressing size requirements as Bio2RDF continues to grow, the reasoning capabilities of triple store platforms will also present a significant performance factor as HyQue must be able to execute SPARQL queries with potentially complex reasoning corresponding to the type hierarchies and axiomatic restrictions of complex ontologies.

### Future Work

We intend to expand the domains to which HyQue is applicable beyond that of the yeast galactose gene network. To achieve this goal, several developments will be required, some of which are currently underway. Successful application to other domains will require scientists to curate their own experimental data and contribute domain specific knowledge bases. Bio2RDF currently contains knowledge from approximately 1600 datasets, and is growing steadily. There are also a number of other online resources that provide data usable by HyQue, including the NCBI and NCBO’s BioPortal. Computational approaches for extracting statements from scientific articles and contributing them to knowledge bases must be developed. There already exists a significant amount of research activity in biological/biomedical text processing and information extraction. There are existing tools such as BANNER [30], BioInfer [31], GeneWays [32], and Textpresso [33], which can be leveraged in developing approaches for extracting knowledge from scientific text and contributing it to HyQue. However, we realize that adapting a text-mining tool developed for one domain area (*e.g.* Textpresso for *C. elegans*) to another scientific domain is a non-trivial and time consuming task. Finally, knowledge about the provenance of biological data is currently missing from Bio2RDF and the HKB. Provenance in this case refers to both the source of data contributed to Bio2RDF (such as who contributed it and when) and the experimental conditions under which experimental data was generated. For example, a yeast two-hybrid assay indicating that two proteins bind is not completely reliable because it does not indicate the stoichiometry of the binding interaction. Provenance is accounted for to some extent by using the Evidence Codes Ontology (ECO), but the granularity of ECO is limited. The Ontology for Biomedical Investigations (OBI) [34] addresses this issue to a greater extent, with classes down to the level of specific assays, types of measurement values, and the relationships between these entities. The addition of such information both retroactively where possible and with future data contributions to Bio2RDF will allow a more sensitive evaluation of hypotheses that can include the quality of the chain of evidence leading to the evaluation.

As the domains HyQue can be applied to increases, so too must the rule sets and scoring approaches that are used to evaluate hypotheses in the context of data and knowledge. Not only will new rule sets need to be created for new domains, but users should also be able to design and contribute their own rule sets for evaluating hypotheses. We intend to develop such a system for HyQue, where rule sets can be provided by users and also contributed to the HyQue archive.

## Conclusions

We have described HyQue, a Semantic Web tool for querying scientific knowledge bases and evaluating biological hypotheses. Currently our system uses a knowledge base that includes background knowledge about the yeast galactose gene network, the proteins and genes that make up this network and the types of biological events these entities are known to participate in. The knowledge base is queried using SPARQL, and queries may include reference to instances or types. Query results are evaluated in reference to the logical structure of a hypothesis to calculate a score indicating the level of support the data lends to the hypothesis. The event-based queries that evaluate hypotheses make use of bio-ontologies (GO, CHEBI, ECO, SO) to retrieve results at varying levels of specificity via subsumption reasoning and entity type checking. Hypotheses as well as the supporting or refuting data are represented in RDF and directly linked to one another allowing scientists to browse from data to hypothesis and *vice versa.* Further information about HyQue and the hypotheses and data used in HyQue are available at http://semanticscience.org/projects/hyque.

## Competing interests

The authors have no competing interests to declare.

## Authors' contributions

AC created the HyQue Knowledge Base, wrote the HyQue hypothesis evaluation code and drafted the manuscript. MD and NS conceived of the study, participated in its design and edited the manuscript. All authors read and approved the final manuscript.
